# Faecal carriage of *Clostridioides difficile* is low among veterinary healthcare workers in the Netherlands

**DOI:** 10.1017/S0950268822000383

**Published:** 2022-02-28

**Authors:** Anouk P. Meijs, Esther F. Gijsbers, Paul D. Hengeveld, Ed J. Kuijper, Cindy M. Dierikx, Sabine C. de Greeff, Engeline van Duijkeren

**Affiliations:** 1 Centre for Infectious Disease Control (CIb), National Institute for Public Health and the Environment (RIVM), Bilthoven, the Netherlands; 2 Department of Medical Microbiology, Leiden University Medical Center, Leiden, the Netherlands

**Keywords:** *Clostridioides difficile*, *Clostridioides difficile* carriage, veterinarians, veterinary healthcare workers

## Abstract

Veterinary healthcare workers are in close contact with many different animals and might be at an increased risk of acquiring *Clostridioides difficile.* In this cross-sectional study, we assessed the prevalence and risk factors of *C. difficile* carriage in Dutch veterinary healthcare workers. Participants provided a faecal sample and filled out a questionnaire covering potential risk factors for *C. difficile* carriage. *C. difficile* culture positive isolates were polymerase chain reaction (PCR) ribotyped and the presence of toxin genes *tcdA*, *tcdB* and *cdtA/cdtB* was determined. Eleven of 482 [2.3%; 95% confidence interval (CI) 1.3–4.0] veterinary healthcare workers were carriers of *C. difficile*. Three persons carried *C. difficile* ribotype 078 (0.6%; 95% CI 0.2–1.8). Risk factors for carriage were health/medication and hygiene related, including poor hand hygiene after patient (animal) contact, and did not include occupational contact with certain animal species. In conclusion, the prevalence of *C. difficile* carriage in veterinary healthcare workers was low and no indications were found that working in veterinary care is a risk for *C. difficile* carriage.

*Clostridioides difficile* is a spore-forming, anaerobic bacterium that can colonise the gastrointestinal tract of both humans and animals. In humans, *C. difficile* can cause infections (*C. difficile infection*, CDI), with symptoms ranging from diarrhoea to severe pseudomembranous colitis. Traditionally, CDI was regarded as a primarily nosocomial disease, but it is now increasingly found in persons outside the healthcare setting [1]. In community-acquired CDI, ribotype 078 (RT078) is emerging as a cause of infection [2]. This type is predominant among pigs and cattle, animals that are frequently found positive for *C. difficile* [3]. Previous research into RT078 has shown that pig farmers and their pigs shared identical *C. difficile* strains and that transmission occurred either via direct contact or via the environment [4, 5]. In a study among persons living near livestock farms in the Netherlands, the prevalence of *C. difficile* carriage was low (1.2%) and 0.2% carried RT 078 [6].

*C. difficile* has also been found in a wide range of animals other than pigs and cattle, including horses, dogs and cats, and the most common strains found in human CDI also occur in cats and dogs [7]. This suggests that household pets could serve as a potential source of *C. difficile* for humans (and vice versa), or that there is a common source of exposure. Indeed Loo *et al*. found that transmission may occur between CDI patients and their household members and domestic pets [8]. However, other studies on *C. difficile* isolates from households have revealed no overlap in ribotypes between dogs or cats and their owners, or between dogs and the household environment [9, 10].

If zoonotic transmission of *C. difficile* occurs, veterinary healthcare workers who are in close contact with diseased and possibly diarrhoeic animals might be at an increased risk of acquiring *C. difficile* and potentially contribute to spreading *C. difficile* in the community. Therefore, the aim of this study is to investigate the prevalence of *C. difficile* carriage and risk factors including occupational contact with different types of animals in veterinary healthcare workers.

The medical ethical committee of the University Medical Center Utrecht reviewed this study and granted it an official exemption for approval under the medical research involving human subjects act (WMO) (number 18-389/C). This study is part of the Antibiotic-Resistant Bacteria in Dutch Veterinary healthcare workers study (Dutch acronym: AREND), in which the presence of ESBL-producing *Escherichia coli* and *Klebsiella pneumoniae*, colistin-resistant *E. coli* and *K. pneumoniae*, and *C. difficile* was determined in persons working in veterinary healthcare. Veterinary personnel (aged 18 years or older) was recruited between August 2018 and March 2019, through flyers sent to veterinary clinics, articles and recruitment at a veterinary conference (KNMvD voorjaarsdagen 2018). All participants signed an informed consent form. Participants sent in a faecal sample collected at home and completed a web-based questionnaire covering potential risk factors for *C. difficile* carriage (Supplementary material). To avoid clustering, participants working in the same clinic were assigned to participate in different months.

Faecal samples were sent to the laboratory by regular mail and upon arrival were either processed the same day or stored at 4 °C for up to 2 days. *C. difficile* was cultured by suspending approximately 1 g of faeces in 9 ml of *C. difficile* enrichment modified broth (Mediaproducts) with C.D.M.N. Selective Supplement (Oxoid) and incubated at 37 °C for 10–15 days under anaerobic conditions. The suspension was inoculated onto ChromID *C. difficile* agar (bioMérieux) directly, as well as following ethanol shock and incubated for 2–5 days under anaerobic conditions. A maximum of three suspected colonies per person were selected for further testing. Bacterial species were confirmed using Matrix-Assisted Laser Desorption/Ionisation Time-Of-Flight Mass Spectrometry (MALDI-TOF MS) (Bruker). Subsequently, *C. difficile* positive isolates were genetically identified as *C. difficile* by polymerase chain reaction (PCR) for the presence of the *gluD* gene [11]. Further *C. difficile* characterisation was performed by PCR ribotyping and by determining the presence of toxin A (*tcdA)*, toxin B (*tcdB)* and the binary toxin (*cdtA/cdtB*) genes [12, 13].

Prevalence of *C. difficile* carriage with 95% confidence intervals (CIs) was determined with the Wilson method [14]. Using univariable logistic regression analysis, crude odds ratios (ORs) with 95% CIs were calculated to study potential risk factors for *C. difficile* carriage. A *P*-value < 0.05 was used to determine significance. Analyses were performed using SAS version 9.4 (SAS Institute Inc., Cary, NC, USA).

Of 515 veterinary healthcare workers that signed the informed consent form, 482 (93.6%) returned the faecal sample and completed the questionnaire. The median age of participants was 38 years (range 20–70 years), and 84.9% were female. The participants worked in veterinary clinics located in 310 different postal code areas. The prevalence of *C. difficile* carriage was 2.3% (11/482; 95% CI 1.3–4.0). Three persons carried *C. difficile* RT078 (prevalence 0.6%; 95% CI 0.2–1.8), see Table 1. Other ribotypes with toxin genes *tcdA* and *tcdB* were found in five participants (006, 046, 351 and two unidentified ribotypes that did not match any isolate in the established database). Three persons carried ribotypes without toxin genes (009, 039 and one unidentified ribotype). The three persons carrying RT078 all worked in different postal code areas. Two were veterinarians frequently working with companion animals, and one also worked with horses. The third person was a veterinary assistant who indicated not to have frequent animal contact at work but had non-occupational contact with pigs in the last 4 weeks, and had a partner who was a pig farmer. All three held animals at home, including dogs, cats and horses. Potential non-work-related risk factors that were present in these persons were having a young child going to day care (*n* = 1), use of proton pump inhibitors (PPI) or antacids due to acid reflux (*n* = 2) and use of antibiotics in the past 6 months (*n* = 1). More characteristics, including those of persons carrying other *C. difficile* strains, are shown in Table 1.

**Table 1.** Characteristics of veterinary healthcare workers who were carrier of *Clostridioides difficile*

Veterinary healthcare worker ID	1	2	3	4	5	6	7	8	9	10	11
	Toxigenic ribotypes								Non-toxigenic ribotypes		
PCR ribotype	078	078	078	006	046	351	UNK	UNK	009	039	UNK
*tcdA*	+	+	+	+	+	+	+	+	−	−	−
*tcdB*	+	+	+	+	+	+	+	+	−	−	−
*cdtA/cdtB*	+	+	+	−	−	−	−	−	−	−	−
Sex	Female	Female	Female	Female	Female	Female	Female	Female	Female	Female	Female
Age category (years)	50–59	30–39	30–39	18–29	30–39	18–29	18–29	30–39	30–39	18–29	40–49
Has children (<4 years) attending day-care	No	Yes	No	No	No	No	No	No	Yes	No	No
Profession	Veterinarian	Veterinary assistant	Veterinarian	Veterinary technician	Veterinarian	Veterinarian	Veterinary technician	Veterinarian	Veterinary technician	Veterinarian	Veterinary technician
No. of animal contact hours at work per week	20	0	30	13	20	20	16	32	10	28	5
Frequent animal contact at work^a^	Dog, cat, rabbit/rodent^b^	None	Dog, cat, rabbit/rodent^b^, bird, horse	Dog, cat, rabbit/rodent^b^, alpaca	Dog, cat	Cattle, sheep, goat	Dog, cat, rabbit/rodent^b^, bird, chicken	Dog, cat, rabbit/rodent^b^, bird	Dog, cat, rabbit/rodent^b^	Dog, cat, rabbit/rodent^b^	Dog, cat
Work-related animal contact in last 4 weeks	Dog, cat, rabbit/rodent^b^	Dog, chicken, horse	Dog, cat, rabbit/rodent^b^, bird, horse	Dog, cat, rabbit/rodent^b^, alpaca	Dog	Cattle, sheep, goat	Dog, cat, rabbit/rodent^b^, bird, chicken	Dog, cat, rabbit/rodent^b^, bird, chicken	Dog, cat, rabbit/rodent^b^	Dog, cat, rabbit/rodent^b^	Dog, cat, cattle
Frequent work activities with companion animals^a^	Consultations, surgical proc.	None	Consultations, home visits, surgical proc., dental care, cleaning of housing, shaving/grooming	Dental care, cleaning of housing	Consultations, shaving/grooming	None	Consultations, surgical proc., cleaning of housing	Consultations, cleaning of housing, shaving/grooming	Consultations, surgical proc., cleaning of housing	Consultations, surgical proc., dental care, cleaning of housing	None
Frequent work activities with livestock^a^	None	None	None	None	None	Farm/home visits, surgical proc.	None	None	None	None	Farm/home visits
Frequent work activities with horses^a^	None	None	Farm/home visits	None	None	None	None	None	None	None	None
Work-related farm visits in last 4 weeks	No	No	No	No	No	Yes, cattle, sheep, goats, petting zoo	No	No	No	No	Yes, cattle
Household member/partner has profession with animal contact	No	Yes, farmer	No	No	No	Yes, veterinarian	No	No	Yes, veterinarian	Yes, veterinary technician	No
Owns a pet or hobby farm animal	Yes, dog, cat	Yes, dog, rabbit/rodent^b^, horse	Yes, cat	No	Yes, cat	No	Yes, rabbit/rodent^b^, bird	No	No	Yes, dog, cat, rabbit/rodent^b^, bird	Yes, dog, cat, chicken, reptile
Non-occupational animal contact in last 4 weeks	Yes, dog, cat	Yes, dog, rabbit/rodent^b^, pig, horse	Yes, dog, cat, horse	Yes, dog, rabbit/rodent^b^, alpaca	Yes, dog, cat, rabbit/rodent^b^	Yes, dog, horse	Yes, dog, cat, rabbit/rodent^b^, bird, chicken	Yes, dog, cat, horse	Yes, dog, cat, bird, pig	Yes, dog, cat, rabbit/rodent^b^, bird	No
Hospitalised in Dutch hospital in last 6 months	No	Yes	No	No	No	No	No	No	No	No	No
PPI or antacid use in last 6 months	No	Yes	Yes	No	No	No	No	Yes	No	No	No
Antibiotic use											
Last 6 months	No	Yes	No	No	Yes	No	No	No	No	Yes	No
Last 3 months	No	Yes	No	No	Yes	No	No	No	No	Yes	No
Medication use in last 6 months^c^	Antihypertensive agents	No	Oral contraceptives, depression meds	Oral contraceptives, depression meds	Oral contraceptives	No	Oral contraceptives	No	Sleeping pills/tranquilizers	Oral contraceptives, depression meds	No
Stomach and/or bowel disease^d^	No	Acid reflux	Acid reflux	No	No	No	No	Acid reflux	No	No	No
Stomach and/or bowel complaints last 4 weeks^e^	No	Yes	No	Yes	No	Yes	Yes	Yes	No	Yes	Yes
Travel in last 6 months	No	Western Europe	Southern and Eastern Europe	Northern Europe	Northern Africa, Western Europe	Southern, Western and Northern Europe	Northern Europe	Southern Europe	No	No	Southern Europe
Diet without meat	No	No	No	No	Yes	No	No	No	No	Yes	No

*tcdA*, toxin A gene; *tcdB*, toxin B gene; *cdtA/cdtB*, binary toxin genes; PPI, proton pump inhibitor; proc., procedures; UNK, unknown.

a Weekly or more often.

b Rabbit, Guinea pig, hamster, rat and/or mouse.

c Including: ADHD medication, oral contraceptives, medication for depression, sleeping pills/tranquilizers, antidiabetic agents, antihypertensive agents, chemotherapy, statins, laxatives.

d Including: gastric mucosal irritation, acid reflux, gastric cancer, colon polyps, colon cancer, irritable bowel syndrome, Crohn's disease, ulcerative colitis, coeliac disease.

e Including: vomiting, nausea, abdominal pain or cramps, mucus or blood in the stool, pale stool, diarrhoea (≥3 times a day).

The results of the univariate risk factor analysis for *C. difficile* carriage are shown in Supplementary material, Table S1. Pig contact (not work related) in the past 4 weeks was the only statistically significant animal-related risk factor (OR 6.8; 95% CI 1.3–34.0). Several hygiene-related factors were associated with an increased risk, including almost never washing hands after patient contact (OR 12.7; 95% CI 1.2–129.2) and poor hygiene practices at home: regularly/sometimes washing hands before food preparation (OR 5.4; 95% CI 1.1–25.6); almost never washing hands after toilet use (OR 7.3; 95% CI 1.3–40.8); and not changing the kitchen dishcloth on a daily basis (OR 8.3; 95% CI 1.1–65.0). Other risk factors were health and medication-related: having acid reflux (OR 4.2; 95% CI 1.1–16.3) and using medication for depression (such as venlafaxine, lithium and monoamine oxidase inhibitors) (OR 10.0; 95% CI 2.4–41.0).

The prevalence of *C. difficile* carriage of 2.3% (95% CI 1.3–4.0) in veterinary healthcare workers was not significantly higher compared to the prevalence of 1.2% (95% CI 0.9–1.7; *n* = 30/2432) that was found in a large Dutch population study among persons living in a rural area with a high density of livestock farms in 2014–2015 [6]. It was lower than the prevalence of 5.1% (95% CI 3.8–6.9) in 765 stool samples of a population of asymptomatic patients with significant comorbidity and medication use on admission to Dutch hospitals [15]. All carriers were female, which was most likely caused by an overrepresentation (85%) of female participants. The majority of *C. difficile* positive isolates (72.7%; *n* = 8/11) contained a toxigenic variant. This is comparable to the distribution of toxigenic/non-toxigenic variants in the paper by Zomer *et al*. (70.0%; *n* = 21/30) [6]. RT078 was the most prevalent ribotype (*n* = 3; 27.3%), while it was the second most prevalent type in the aforementioned study, after RT014. RT014 was not detected in the present study. In the Dutch sentinel surveillance of CDI in 2019–2020 RT014 was the most frequently isolated ribotype (18.1%), whereas RT078 accounted for 8.7% of CDI [16].

RT078 has been reported as the predominant type in pigs in the Netherlands [7], but only a minority of the veterinary workers had frequent occupational contact with pigs (*n* = 19; 3.9%), and only one of the three RT078 *C. difficile* positives had (non-occupational) contact with pigs. We found an association between *C. difficile* carriage and non-occupational contact with pigs, although this was based on only two *C. difficile* positive persons.

To our knowledge, this is the first study that investigated *C. difficile* carriage in veterinary healthcare workers. Most of the participants (>85%) had occupational contact with dogs and cats, and 69% had occupational contact with companion animals only and not with livestock. There are around 2400 veterinary clinics in the Netherlands of which 60% are companion animal clinics, 15% are livestock clinics, 5% are horse clinics, and 20% are mixed clinics [17]. The distribution of participants in our study working with companion animals (90%), livestock (23%) and horses (16%) is therefore representative for the country. The exact number of clinics represented in our study is unknown, but personnel from veterinary clinics located in 310 different 4-digit postal code areas were included (from a total of 4070 of these areas in the Netherlands).

*C. difficile* carriage has been described in healthy and diarrhoeic companion animals [3]. Furthermore, studies in veterinary clinics demonstrated *C. difficile* being present in companion animals visiting the clinic as well as on the clinic's surfaces, suggesting potential transmission at the clinic [18, 19]. We found an increased risk of *C. difficile* carriage for poor hand hygiene after patient contact, which could indicate a potential route of exposure via patients. However, since the prevalence in veterinary healthcare workers was low, the risk of transmission was likely very small.

Although clinical and epidemiological risk factors of CDI have been studied frequently [20], studies on risk factors of *C. difficile* carriage are still scarce, especially for community-acquired carriage [21]. Known risk factors of *C. difficile* carriage in the healthcare setting include recent hospitalisation and the use of specific medication, such as immunosuppressant, antibiotics and PPI or H_2_ blockers [21]. Among predominantly healthy young infants, the risk was increased in infants with a pet dog [22], and in the general population antibiotic use was previously identified as a risk factor [6]. We found a non-significant association between antibiotic use and *C. difficile* carriage, presumably due to the small number of participants that were *C. difficile* positive. Furthermore, having acid reflux (but not the use of PPI or antacids) as well as the use of medication for depression was associated with a higher risk of *C. difficile* carriage. This association that was found with certain types of medication could be explained by the influence that they have on the microbiome [21, 23], and both CDI and carriage have been associated with an altered microbiome and a decreased bacterial diversity in the gut [24].

This study had some limitations. First, due to the small number of *C. difficile* positive participants, estimates of potential risk factors are weak. To obtain robust insights into general risk factors for *C. difficile* carriage, large population studies are needed. Second, we did not include a control group of persons without occupational animal contact, since we were mainly interested in specific occupational risk factors in veterinary healthcare. The prevalence in veterinary healthcare workers was compared to the prevalence that was found in a large Dutch population study performed 4 years earlier [6]. Finally, the risk factors assessed in this study are based on self-reporting, it is possible that some exposures were under- or overreported due to recall bias.

In conclusion, the prevalence of *C. difficile* carriage in veterinary healthcare workers was low and no indications were found that working in veterinary care increased the risk of *C. difficile* carriage.

## Data Availability

The data that support the findings of this study are available on request from the corresponding author. The data are not publicly available due to their containing information that could compromise the privacy of research participants.

## Supplementary material

For supplementary material accompanying this paper visit http://doi.org/10.1017/S0950268822000383. click here to view supplementary material
