# Supplementary material for: Faecal carriage of Clostridioides difficile is low among veterinary healthcare workers in the Netherlands
Source: Epidemiol Infect. 2022 Feb 28;150:e63. doi: 10.1017/S0950268822000383 (PMC8931804; doi:10.1017/S0950268822000383)
Supplement: Supplementary file 1 [file S0950268822000383sup001.docx]

*Epidemiology and Infection*

Faecal carriage of *Clostridioides difficile* is low among veterinary healthcare workers in the Netherlands

Anouk P. Meijs, Esther F. Gijsbers, Paul D. Hengeveld, Ed J. Kuijper, Cindy M. Dierikx, Sabine C. de Greeff, Engeline van Duijkeren

**Supplementary Material**

- Questionnaire
- Supplementary Table S1

**AREND study questionnaire**

**Background information:**

This questionnaire contains questions regarding your work, contact with animals, health, leisure activities, food and hygiene. It takes about 15-25 minutes to answer all questions. The questionnaire is anonymous, and your data will be treated confidentially. Most questions can be answered by checking the box next to the answer that applies (most) to you. Your answers will be linked to the results of your faecal sample, for which you have received a collection package.

**Thank you very much for your cooperation!**

If you have any questions or comments, please contact us by sending an email to the address below:

| **A. General**   1. **What is your sex?**  - Male - Female |
| --- |
| 1. **What are the 4 digits of your postal code?**   ⬜⬜⬜⬜ |
| 1. **What is your country of birth?**  - Netherlands - Other country, namely …..………….……………… |
| 1. **What is your date of birth?**   ⬜⬜⬜⬜ |
| 1. **Do you have children (aged below 4 years) who go to childcare or a day care centre?**  - No - Yes |
| 1. **What is your current profession?** *Multiple answers possible*  - Practising veterinarian - Veterinary assistant - Veterinary technician - Animal caretaker - Animal physiotherapist - Veterinary medicine student (intern) - Other, namely ................................. |
| 1. **What type of animals are treated in the veterinary clinic where you work?** *Multiple answers possible*  - Companion animals - Livestock - Horses - Other, namely …............................... |
| 1. **What are the 4 digits of the postal code(s) of the veterinary clinic(s) where you work?**   ⬜⬜⬜⬜  ⬜⬜⬜⬜  ⬜⬜⬜⬜ |
| 1. **In which province(s) do you work at the moment (incl. farm visits)?** *Multiple answers possible*  - Groningen - Friesland - Drenthe - Flevoland - Noord-Holland - Zuid-Holland - Overijssel - Gelderland - Utrecht - Zeeland - Noord-Brabant - Limburg |

| **B. Work activities and professional contact with animals** |
| --- |
| 1. **On average, how many hours per week do you work in veterinary healthcare?**   **On average, how many hours per week do you have professional contact with animals?**  Number of working hours a week: ................................................  Number of animal-contact hours a week: ............................................ |
| 1. **How many years have you been doing this work?**   ⬜⬜ |
| 1. **How often do you have professional contact with the following animal species or their faeces?** *Contact is defined as: touching the skin, fur or faeces/manure, and cleaning of the housing or stables*  \|  \| (Almost) daily \| Weekly \| Monthly \| (Almost) never \| \| --- \| --- \| --- \| --- \| --- \| \| Dogs \|  \|  \|  \|  \| \| Cats \|  \|  \|  \|  \| \| Rabbits/guinea pigs/hamsters \|  \|  \|  \|  \| \| Mice/rats \|  \|  \|  \|  \| \| Birds \|  \|  \|  \|  \| \| Cattle \|  \|  \|  \|  \| \| Sheep \|  \|  \|  \|  \| \| Goats \|  \|  \|  \|  \| \| Chicken \|  \|  \|  \|  \| \| Other poultry \|  \|  \|  \|  \| \| Pigs \|  \|  \|  \|  \| \| Horses/pony’s \|  \|  \|  \|  \| \| Donkeys \|  \|  \|  \|  \| \| Reptiles \|  \|  \|  \|  \| \| Other, namely ......................  . \|  \|  \|  \|  \| |
| 1. **How often do you professionally carry out the following tasks?**  \|  \| (Almost) daily \| Weekly \| Monthly \| (Almost) never \| \| --- \| --- \| --- \| --- \| --- \| \| **Companion animals** \|  \|  \|  \|  \| \| Consultations \|  \|  \|  \|  \| \| Home visits \|  \|  \|  \|  \| \| Surgical procedures \|  \|  \|  \|  \| \| Dental cleaning/care \|  \|  \|  \|  \| \| Cleaning animal housing \|  \|  \|  \|  \| \| Shaving/grooming \|  \|  \|  \|  \| \| **Livestock** \|  \|  \|  \|  \| \| Farm/home visits \|  \|  \|  \|  \| \| Surgical procedures \|  \|  \|  \|  \| \| Cleaning out stables \|  \|  \|  \|  \| \| Inspection of live animals for transport/slaughter \|  \|  \|  \|  \| \| Inspection of meat/ carcasses in slaughterhouse \|  \|  \|  \|  \| \| **Horses** \|  \|  \|  \|  \| \| Outpatient clinic \|  \|  \|  \|  \| \| Farm/home visits \|  \|  \|  \|  \| \| Surgical procedures \|  \|  \|  \|  \| \| Dental cleaning/care \|  \|  \|  \|  \| \| Cleaning out stables \|  \|  \|  \|  \| \| Brushing/grooming \|  \|  \|  \|  \| |
| 1. **Which animal species or their faeces have you had professional contact with in the last 4 weeks?** *Multiple answers possible*  - Dogs - Cats - Rabbits/guinea pigs/hamsters - Mice/rats - Birds - Cattle - Sheep - Goats - Chicken - Other poultry - Pigs - Horses/pony’s - Donkeys - Reptiles - Other, namely ............................................................................ |
| 1. **Have you visited animal farms in the last 4 weeks?** (Only if A7 = Livestock or Other)  - No 🡪 *go to part* **C** - Yes |
| 1. **What type of farms have you visited in the last 4 weeks?** (Only if A7 = Livestock of Other) *Multiple answers possible*  - Cattle, beef - Cattle, milk - Cattle, other .................... - Sheep - Goats - Poultry, broilers - Poultry, laying hens - Poultry, other .......................... - Pigs, porkers - Pigs, meat - Pigs, other .............................. - Other (such as mixed animal farm, petting zoo), namely ........................................................ |

| **C. Personal protection at work** |
| --- |
| 1. **How often do you use gloves during the following tasks?**  \|  \| (Almost) never \| Sometimes \| Regularly \| (Almost) always \| Not applicable \| \| --- \| --- \| --- \| --- \| --- \| --- \| \| General examination \|  \|  \|  \|  \|  \| \| Surgical procedures \|  \|  \|  \|  \|  \| \| Dental treatments \|  \|  \|  \|  \|  \| \| Rectal exploration \|  \|  \|  \|  \|  \| \| Treatment of anal glands \|  \|  \|  \|  \|  \| \| Birth/delivery \|  \|  \|  \|  \|  \| \| Cleaning of dirty wounds \|  \|  \|  \|  \|  \| \| Inspection of live animals for transport/slaughter \|  \|  \|  \|  \|  \| \| Other, namely ........................... \|  \|  \|  \|  \|  \| |
| 1. **When you have had contact with patients, how often do you wash or sanitize your hands before taking a break or going home?**  - (Almost) never - Sometimes - Regularly - (Almost) always - Not applicable |
| 1. **Are you involved in cleaning the animal housing and/or cleaning out the stables at work?**  - No 🡪 *Go to* **C5** - Yes |
| 1. **What type of protection do you wear when cleaning the animal housing and/or cleaning out the stables?**  \|  \| (Almost) never \| Sometimes \| Regularly \| (Almost) always \| \| --- \| --- \| --- \| --- \| --- \| \| Gloves \|  \|  \|  \|  \| \| Face mask \|  \|  \|  \|  \| \| Lab coat \|  \|  \|  \|  \| \| Company clothing (overalls etc) \|  \|  \|  \|  \| \| Boots/clogs \|  \|  \|  \|  \| \| Other, namely .................... \|  \|  \|  \|  \| |
| 1. **How often is the treatment table cleaned?** (Only if A7 = Companion animals or Other)  - After every patient - A few times a day - At the end of the day - Not applicable |
| 1. **Which cleaning agent is used to clean the treatment table?** (Only if A7 = Companion animals or Other) *Multiple answers possible*  - Water - Disinfectant - Soap - Dry cloth - Not applicable - Other, namely............................................................................. |

| **D. Profession of partner/household member** |
| --- |
| 1. **Do you have a partner/household member who has professional contact with animals (e.g., veterinarian, veterinary assistant/technician, livestock farmer, employee at riding school or farm, slaughterhouse employee)?**  - No 🡪 *go to* **D4** - Yes |
| 1. **What profession does he/she have?**   ...................................................................................................................... |
| 1. **Which animal species does he/she have professional contact with?** *Multiple answers possible*  - Dogs - Cats - Rabbits/guinea pigs/hamsters - Mice/rats - Birds - Cattle - Sheep - Goats - Chicken - Other poultry - Pigs - Horses/pony’s - Donkeys - Reptiles - Other, namely ............................................................................. |
| 1. **Do you have a partner/household member who works in the healthcare sector and who has contact with patients/clients/residents (e.g. social worker, nurse, doctor)?**  - No 🡪 *Go to part* **E** - Yes |
| 1. **What profession does he/she have?**   ...................................................................................................................... |

| **E. Animal contact at home** |
| --- |
| 1. **Do you live on an animal farm?**  - No 🡪 *Go to* **E3** - Yes |
| 1. **On what type of farm do you live?** *Multiple answers possible*  - Dairy farm (cattle) - Veal calf farm - Goat farm - Sheep farm - Pig farm (breeding sows, fattening pigs, etc.) - Poultry farm with laying hens - Poultry farm with broilers - Poultry farm with turkeys - Poultry farm with ducks - Riding school or stud farm - Farm with (*fill in animal type*) ................................................... |
| 1. **Do you keep companion animals or livestock around your home?**  - No 🡪 *Go to* **E6** - Yes |
| 1. **Which animal species do you keep around your home?** *Multiple answers possible*  - Dogs - Cats - Rabbits/guinea pigs/hamsters - Mice/rats - Birds - Cattle - Sheep - Goats - Chicken - Other poultry - Pigs - Horses/pony’s - Donkeys - Reptiles - Other, namely ............................................................................. |
| 1. **How many animals do you have?**   Number of dogs: ⬜⬜⬜  Number of cats: ⬜⬜⬜  Number of rabbits/guinea pigs/hamsters: ⬜⬜⬜  Number of mice/rats: ⬜⬜⬜  Number of birds: ⬜⬜⬜  Number of cattle: ⬜⬜⬜  Number of sheep: ⬜⬜⬜  Number of goats: ⬜⬜⬜  Number of chickens: ⬜⬜⬜  Number of other poultry: ⬜⬜⬜  Number of pigs: ⬜⬜⬜  Number of horses/pony’s: ⬜⬜⬜  Number of donkeys: ⬜⬜⬜  Number of reptiles: ⬜⬜⬜  Number of other animals: ⬜⬜⬜ |
| 1. **Do you ever feed your animals raw meat?**  - No - Yes |
| 1. **Have you had direct contact with companion animals or livestock in the last 4 weeks (non-professional!)?** *These can be your own animals, someone else’s animals or at a petting zoo. Direct contact is defined as: touching the animal or its faeces/manure.*  - No 🡪 *Go to part* **F** - Yes |
| 1. **Which animal species have you had direct contact with in the last 4 weeks (non-professional!)?** *These can be your own animals, someone else’s animals or at a petting zoo. Direct contact is defined as: touching the animal or its faeces/manure.*   *Multiple answers possible*   - Dogs - Cats - Rabbits/guinea pigs/hamsters - Mice/rats - Birds - Cattle - Sheep - Goats - Chicken - Other poultry - Pigs - Horses/pony’s - Donkeys - Reptiles - Other, namely ............................................................................. |

| **F. Health and medication use** |
| --- |
| 1. **Have you been hospitalized in the last 6 months in a Dutch hospital (1 overnight stay or more)?**  - No 🡪 *go to* **F3** - Yes |
| 1. **Have you been hospitalized in the last 6 months in a foreign hospital (1 overnight stay or more)?**  - No - Yes, (*please fill in the name of the country*) …………………………………………………………….. |
| 1. **Has any of your family members been admitted to a hospital or nursing home in the last 6 months (1 overnight stay or more)?**  - No - Yes |
| 1. **Have you used proton pump inhibitors or antacids in the last 6 months?** E.g. omeprazol, pantozol, ranitidine, pantoprazol, lansoprazol, rabeprasol, esomeprazol  - No - Yes, (*please fill in the name of the drug)* ………………………………………………………………………… |
| 1. **Have you used antibiotics in the last 6 months?** E.g. feneticillin Broxil®, flucloxazillin Floxapen®, amoxicillin, amoxicillin-clavulanic acid Augmentin®  - No → *go to* **F11** - Yes |
| 1. **How many courses of antibiotic treatment have you used in the last 6 months?**   ⬜⬜⬜ |
| 1. **What was the name of the antibiotic you used last?**  - Amoxicillin - Amoxicillin-clavulanic acid - Cefaclor - Cefalexin - Cefixime - Ceftibuten - Ceftriaxone - Cefuroxime axetil, cefuroxime - Feneticillin - Flucloxacillin - Metronidazole - Penicillin - Clindamycin - Ciprofloxacin - Levofloxacin - I do not now - Other, namely ……………………………………………………………………………… |
| 1. **When did you use antibiotics for the last time?**  - I still use it - Less than 3 months ago - 3-6 months ago |
| 1. **In addition to those already mentioned, which antibiotics have you taken in the last 6 months?**  - Amoxicillin - Amoxicillin-clavulanic acid - Cefaclor - Cefalexin - Cefixime - Ceftibuten - Ceftriaxone - Cefuroxime axetil, cefuroxime - Feneticillin - Flucloxacillin - Metronidazole - Penicillin - Clindamycin - Ciprofloxacin - Levofloxacin - I do not now - Other, namely ……………………………………………………………………………… |
| 1. **Have you used any of the following medication in the last 6 months?** *Multiple answers possible*  - Statins (e.g. simvastatin, pravastatin, atorvastatin) - Laxatives (e.g. colophort, endofalk, movicolon, molaxole) - Antihypertensive agents (e.g. betaxolol, valsartan, amlodipine) - ADHD medication (e.g. ritalin) - Medication for depression (e.g. venlafaxine, lithium, MAO inhibitors) - Sleeping pills and tranquilizers (e.g. diazepam, oxazepam, temazepam) - Antidiabetics (e.g. metformin, glimepiride, levemir, novorapid) - Contraceptive pill (for women) - Chemotherapy - I prefer not to answer - None of the above |
| 1. **Do you suffer from any of the following stomach and/or bowel diseases?**  - Gastric mucosa irritation - Acid reflux - Stomach cancer - Colon polyps - Colon cancer - Irritable bowel syndrome - Crohn’s disease - Ulcerative colitis - Celiac disease - None of the above |
| *Please note: the following questions refer to the last 4 weeks* |
| 1. **Have you had stomach and/or bowel complaints in the last 4 weeks?** *(E.g. vomiting, nausea, abdominal pain, diarrhoea, mucus or blood in the stool)*  - No → *go to part* **G** - Yes |
| 1. **Please tick the complaints that you have had in the last 4 weeks.** *Multiple answers possible*  - Vomiting - Nausea - Abdominal pain/abdominal cramp - Mucus in the stool - Blood in the stool - Light (decoloured) stool - Diarrhoea (at least 3 times a day) - Other, namely .............................................................................. |
| 1. **How many day have you had these complaints in the last 4 weeks?**   ⬜⬜⬜ |

| **G. Leisure activities** |
| --- |
| 1. **Have you been abroad in the past 6 months?**  - No 🡪 *go to* **G3** - Yes |
| 1. **Which countries have you been to in the past 6 months? How long did you stay and when did you return?**  *If you made more than 5 trips, enter the 5 with the longest duration*   **Which country/countries? How long did you stay? Which month did you return** 1…………………………… …… days Month: ……………….........  2…………………………… …… days Month: ……………….........  3…………………………… …… days Month: ……………….........  4…………………………... …… days Month: ……………….........  5…………………………… …… days Month: ………………......... |
| 1. **Have you swum in salt or fresh open water in the past 6 months?**  - No, I have not swum in fresh or salt water - Yes, I swam in salt water (sea) - Yes, I swam in fresh open water (e.g. lake, river, other than in a swimming pool) - Yes, I swam in salt and fresh open water |
| 1. **Have you worked with animal manure in the garden in the past 6 months?**  - No - Yes - I do not know |

| **H. Diet and hygiene** |
| --- |
| 1. **What is your diet with respect to meat and fish?**  - I eat both meat and fish - I don’t eat meat and fish, but I do eat other animal products (such as dairy products and eggs) - I don’t eat animal products (including no dairy products and eggs etc.) - I don’t eat meat, but I do eat fish - I eat meat, but I don’t eat fish - I have another diet, namely .................................................................... |
| 1. **For how many years have you been following this diet?**   ⬜⬜⬜ years |
| 1. **Do you (or the person who cooks in your household) use the same cutting board that is used for cutting raw meat, also for other food during the preparation of the same meal? *Please t***ick the option that you do most often (Note: if you don’t eat meat yourself, it may still be the case that meat is prepared in your household)  - No - Yes, but I turn the cutting board - Yes, but I wash the cutting board before using it for other food items - Yes, I use the same cutting board without washing it - Not applicable (I do not use a cutting board or raw meat is never prepared in my household) - I don’t know |
| 1. **Do you (or the person who cooks in your household) use the same knife that is used for cutting raw meat, also for cutting other food during the preparation of the same meal?** ***Please t***ick the option that you do most often (Note: if you don’t eat meat yourself, it may still be the case that meat is prepared in your household)  - No - Yes, but I wash the knife before using it for other food items - Yes, I use the same knife without washing it - Not applicable - I don’t know |
| 1. **Do you (or the person who cooks in your household) wash your hands before food preparation?**  - (Almost) never - Sometimes - Regularly - (Almost) always |
| 1. **Do you wash your hands after toilet use?**  - (Almost) never - Sometimes - Regularly - (Almost) always |
| 1. **Do you use a dishcloth in the kitchen?**  - No, I never use a dishcloth - I only use disposable products (wipes/tissues) and discard them after use - Yes, I use a clean dishcloth every day - Yes, I use the same dishcloth multiple days per week |

| **On which day did you collect your faecal sample, or on which day do you think you will collect it? *(dd – mm – yyyy)***  ⬜⬜ - ⬜⬜ - ⬜⬜⬜⬜ |
| --- |

**Thank you very much for your participation!**

Supplementary Table S1. Assessment of risk factors of *Clostridioides difficile* carriage in veterinary healthcare workers by univariable logistic regression analysis

| **Determinant** | ***C. difficile* positives (*n=11*)** | |  | ***C. difficile* negatives (*n=471*)** | **OR (95%CI)** | | |
| --- | --- | --- | --- | --- | --- | --- | --- |
|  | **n (%)** | |  | **n (%)** |  |  |  |
| Sex |  |  |  |  | |  |  |
| male | 0 (0) | |  | 73 (15.5) | - | | |
| female | 11 (100) | |  | 398 (84.5) | - | | |
| Age |  |  |  |  | |  |  |
| 18-29 years | 4 (36.4) | |  | 98 (20.8) | 4.16 (0.46-37.91) | | |
| 30-39 years | 5 (45.5) | |  | 169 (35.9) | 3.02 (0.35-26.20) | | |
| 40-49 years | 1 (9.1) | |  | 102 (21.7) | 1.00 (0.06-16.21) | | |
| 50-70 years | 1 (9.1) | |  | 102 (21.7) | Ref. | | |
| Born in the Netherlands | 11 (100) | |  | 460 (97.7) | - | | |
| Has children (<4 years of age) attending day-care | 2 (18.2) | |  | 66 (14.0) | 1.36 (0.29-6.45) | | |
| Urbanisation level |  |  |  |  | |  |  |
| very high (≥2500 addresses/km^2^) | 2 (18.2) | |  | 55 (11.7) | 2.69 (0.44-16.50) | | |
| high/moderate (1000-2500 addresses/ km^2^) | 6 (54.6) | |  | 192 (40.8) | 2.31 (0.57-9.37) | | |
| low/very low (<1000 addresses/ km^2^) | 3 (27.3) | |  | 222 (47.1) | Ref. | | |
| **Occupational animal contact** |  |  |  |  | |  |  |
| Profession |  |  |  |  | |  |  |
| veterinarian | 6 (54.6) | |  | 220 (46.7) | 1.47 (0.41-5.29) | | |
| veterinary technician^1^ | 4 (36.4) | |  | 216 (45.9) | Ref. | | |
| veterinary assistant^2^ | 1 (9.1) | |  | 35 (7.4) | 1.54 (0.17-14.20) | | |
| No. of animal contact hours per week (median; IQR) | 20 (10-28) | |  | 21 (15-30) | 0.96 (0.90-1.02) | | |
| Frequent contact with companion animals^3^ | 9 (81.8) | |  | 426 (90.5) | 0.48 (0.10-2.27) | | |
| Frequent contact with livestock^3^ | 3 (27.3) | |  | 107 (22.7) | 1.28 (0.33-4.89) | | |
| Frequent contact with equines^3^ | 1 (9.1) | |  | 77 (16.4) | 0.51 (0.07-4.06) | | |
| Animal contact with (last 4 weeks) |  |  |  |  | |  |  |
| dogs | 10 (90.9) | |  | 408 (86.6) | 1.54 (0.19-12.27) | | |
| cats | 8 (72.7) | |  | 403 (85.6) | 0.45 (0.12-1.74) | | |
| rabbits/rodents^4^ | 7 (63.6) | |  | 325 (69.0) | 0.79 (0.23-2.73) | | |
| birds | 3 (27.3) | |  | 116 (24.6) | 1.15 (0.30-4.40) | | |
| cattle | 2 (18.2) | |  | 79 (16.8) | 1.10 (0.23-5.20) | | |
| sheep/goats | 1 (9.1) | |  | 72 (15.3) | 0.55 (0.07-4.40) | | |
| poultry | 3 (27.3) | |  | 82 (17.41) | 1.78 (0.46-6.85) | | |
| pigs | 0 (0) | |  | 32 (6.8) | - | | |
| horses | 2 (18.2) | |  | 92 (19.5) | 0.92 (0.19-4.31) | | |
| Farm visits (last 4 weeks) | 2 (18.2) | |  | 70 (14.9) | 1.27 (0.27-6.02) | | |
| Hand washing frequency after patient contact |  |  |  |  | |  |  |
| (almost) always | 5 (45.5) | |  | 317 (67.3) | Ref. | | |
| regularly/sometimes | 5 (45.5) | |  | 145 (30.8) | 2.19 (0.62-7.67) | | |
| (almost) never | 1 (9.1) | |  | 5 (1.1) | 12.68 (1.24-129.24)* | | |
| Household member/partner has profession with animal contact | 4 (36.4) | |  | 74 (15.7) | 3.07 (0.88-10.74) | | |
| **Non-occupational animal contact** |  |  |  |  | |  |  |
| Owning a pet or hobby farm animal | 7 (63.6) | |  | 299 (63.5) | 1.01 (0.29-3.49) | | |
| owning dog(s) | 4 (36.4) | |  | 172 (36.5) | 0.99 (0.29-3.44) | | |
| owning cat(s) | 5 (45.5) | |  | 179 (38.0) | 1.36 (0.41-4.52) | | |
| owning rabbits/rodents^4^ | 3 (27.3) | |  | 82 (17.4) | 1.78 (0.46-6.85) | | |
| owning bird(s) | 2 (18.2) | |  | 32 (6.8) | 3.05 (0.63-14.71) | | |
| owning cow(s) | 0 (0) | |  | 7 (1.5) | - | | |
| owning sheep/goats | 0 (0) | |  | 27 (5.7) | - | | |
| owning poultry | 1 (9.1) | |  | 78 (16.6) | 0.50 (0.06-3.99) | | |
| owning horses | 1 (9.1) | |  | 62 (13.2) | 0.66 (0.08-5.24) | | |
| Animal contact (last 4 weeks) | 10 (90.9) | |  | 439 (93.2) | 0.73 (0.09-5.87) | | |
| dogs | 10 (90.9) | |  | 381 (80.9) | 2.36 (0.30-18.68) | | |
| cats | 7 (63.6) | |  | 335 (71.1) | 0.71 (0.21-2.47) | | |
| rabbits/rodents^4^ | 5 (45.5) | |  | 138 (29.3) | 2.01 (0.60-6.70) | | |
| birds | 3 (27.3) | |  | 49 (10.4) | 3.23 (0.83-12.58) | | |
| cattle | 0 (0) | |  | 37 (7.9) | - | | |
| sheep/goats | 0 (0) | |  | 66 (14.0) | - | | |
| poultry | 1 (9.1) | |  | 65 (13.8) | 0.63 (0.08-4.96) | | |
| pigs | 2 (18.2) | |  | 15 (3.2) | 6.76 (1.34-34.01)* | | |
| horses | 4 (36.4) | |  | 159 (33.8) | 1.12 (0.32-3.89) | | |
| **Health and medication use** |  |  |  |  | |  |  |
| Hospitalized in Dutch hospital (last 6 months) | 1 (9.1) | |  | 15 (3.2) | 3.04 (0.37-25.30) | | |
| Antibiotic use |  |  |  |  | |  |  |
| last 6 months | 3 (27.3) | |  | 84 (17.8) | 1.73 (0.45-6.65) | | |
| last 3 months | 3 (27.3) | |  | 51 (10.8) | 3.09 (0.79-12.01) | | |
| PPI or antacid use (last 6 months) | 3 (27.3) | |  | 61 (13.0) | 2.52 (0.65-9.76) | | |
| Has acid reflux | 3 (27.3) | |  | 39 (8.3) | 4.16 (1.06-16.31)* | | |
| Medication use (last 6 months) |  |  |  |  | |  |  |
| ADHD medication | 0 (0) | |  | 5 (1.1) | - | | |
| oral contraceptives | 5 (45.5) | |  | 120 (25.5) | 2.43 (0.73-8.11) | | |
| medication for depression | 3 (27.3) | |  | 17 (3.6) | 9.99 (2.43-41.03)* | | |
| sleeping pills/tranquilizers | 1 (9.1) | |  | 35 (7.4) | 1.24 (0.16-9.99) | | |
| antihypertensive agents | 1 (9.1) | |  | 21 (4.5) | 2.14 (0.26-17.49) | | |
| statins | 0 (0) | |  | 7 (1.5) | - | | |
| laxatives | 0 (0) | |  | 10 (2.1) | - | | |
| Stomach and/or bowel complaints (last 4 weeks) | 7 (63.6) | |  | 181 (38.4) | 2.80 (0.81-9.71) | | |
| **Leisure activities** |  |  |  |  | |  |  |
| Travel (last 6 months) |  |  |  |  | |  |  |
| no travel, travel to Western/Northern Europe, North America, Australia or New Zeeland | 6 (54.6) | |  | 259 (55.0) | Ref. | | |
| travel to Southern/Eastern Europe | 4 (36.4) | |  | 145 (30.8) | 1.19 (0.33-4.29) | | |
| travel to Africa, Asia or Latin America | 1 (9.1) | |  | 67 (14.2) | 0.64 (0.08-5.44) | | |
| **Diet and hygiene** |  |  |  |  | |  |  |
| Diet without meat | 2 (18.2) | |  | 34 (7.2) | 2.86 (0.59-13.75) | | |
| Hand washing frequency before food preparation |  |  |  |  | |  |  |
| (almost) always | 2 (18.2) | |  | 253 (53.7) | Ref. | | |
| regularly/sometimes | 8 (72.7) | |  | 188 (39.9) | 5.38 (1.13-25.64)* | | |
| (almost) never | 1 (9.1) | |  | 30 (6.4) | 4.22 (0.37-47.90) | | |
| Hand washing frequency after toilet use |  |  |  |  | |  |  |
| (almost) always | 5 (45.5) | |  | 274 (58.2) | Ref. | | |
| regularly/sometimes | 4 (36.4) | |  | 182 (38.6) | 1.20 (0.32-4.54) | | |
| (almost) never | 2 (18.2) | |  | 15 (3.2) | 7.31 (1.31-40.80)* | | |
| Uses dishcloth for more than 1 day | 10 (90.9) | |  | 258 (54.8) | 8.25 (1.05-64.97)* | | |

ADHD: Attention-deficit/hyperactivity disorder, CI: confidence interval, IQR: interquartile range, OR: odds ratio, PPI: proton pump inhibitor, Ref.: reference.

* p-value <0.05

^1^ This group also includes animal physiotherapists. Veterinary technicians are legally authorized to perform actions such as administering injections and to examine the animals.

^2^ This group also includes animal caretakers. Veterinary assistants perform more administrative tasks compared to technicians.

^3^ Weekly or more often

^4^ Rabbits, Guinea pigs, hamsters, rats and/or mice
